# Integrated Transcriptomic and Network Analysis Identifies Candidate Kinase-Mediated Thyroid-Associated Targets of Withaferin A

**DOI:** 10.3390/ijms27198926

**Published:** 2026-10-08

**Authors:** Ejlal Mohamed Abdullah, Alaa Alnoor Alameen, Nouf Omar AlAfaleq, Mona Shujaa Alharbi

**Affiliations:** 1 Department of Biochemistry, College of Science, King Saud University, P.O. Box 2455, Riyadh 11451, Saudi Arabia; nalafaleg@ksu.edu.sa (N.O.A.);; 2 Department of Pharmacology and Toxicology, College of Pharmacy, King Saud University, P.O. Box 2457, Riyadh 11451, Saudi Arabia; aalameen1.c@ksu.edu.sa

**Keywords:** withaferin A, hypothyroidism, transcriptomics, kinase enrichment, molecular docking, molecular dynamics

## Abstract

Withaferin A (WA), a bioactive steroidal lactone derived from *Withania somnifera*, has been reported to exhibit diverse pharmacological activities, including anti-inflammatory and metabolic regulatory effects. Although previous studies suggest a potential link between WA and thyroid-related molecular pathways, the molecular mechanisms underlying these effects remain incompletely understood. Here, we employed a comprehensive computational approach to explore WA-responsive molecular changes potentially relevant to thyroid-associated processes. RNA-sequencing data from WA-treated MCF-10A human mammary epithelial cells (GSE158085) were analysed to identify differentially expressed genes (DEGs). Because thyroid-specific WA transcriptomic datasets are not currently available, the DEGs were intersected with curated hypothyroidism-associated genes to generate a cross-tissue, exploratory set of candidate genes. The genes that overlapped were used to construct protein–protein interaction networks, prioritize high-centrality candidate genes, and conduct enrichment analyses for transcription factors and kinases. Further analysis of selected upstream kinases was performed using molecular docking and molecular dynamics simulations with WA. A total of 1458 differentially expressed genes (DEGs) were identified, 10 of which overlapped with hypothyroidism-associated genes. Network analysis identified candidate interactions among the overlapping genes, while higher-confidence analysis retained four connected genes (PTPN22, STAT4, DUOX2, and DUOXA2). Transcription factor enrichment identified ERG, SUZ12, NANOG, TCF3, RUNX1, and TP63 as potential upstream regulators, while kinase enrichment identified candidate upstream kinases associated with the predicted regulatory network. Based on enrichment evidence, structural availability, and literature-supported biological relevance, MAPK14 (p38α), AKT1, and MAPK8 (JNK1) were selected for molecular docking and molecular dynamics simulations. Structural analyses suggested potential interactions between WA and the selected kinases, with target-specific dynamic behaviour across the simulated systems. Collectively, the findings highlight WA-responsive genes and predicted gene–TF–kinase relationships that may be relevant to thyroid-associated molecular processes. However, these associations are hypothesis-generating and provide a basis for further investigation in thyroid-derived experimental models.

## 1. Introduction

Ashwagandha (*Withania somnifera*), commonly known as Indian ginseng or winter cherry, is a medicinal plant widely used in traditional medicine for its adaptogenic properties, particularly in helping the body manage stress. The roots and leaves of this small shrub are commonly processed into extracts and powders [1]. Ashwagandha contains a diverse range of bioactive compounds, including withanolides, alkaloids, steroidal lactones, flavonoids, and other phytochemicals. Among these, withanolides are considered the principal active constituents, with Withaferin A (WA) recognized as one of the most biologically active compounds, followed by Withanolide A and Withanone [2,3]. WA has attracted particular interest because of its reported anti-inflammatory, anti-proliferative, and anticancer properties [4,5,6]. In addition to these pharmacological effects, growing evidence suggests that Ashwagandha may influence endocrine function, particularly thyroid hormone regulation. Clinical and preclinical studies indicate that its supplementation may alter triiodothyronine (T3) and thyroxine (T4) levels in some individuals, potentially through the activity of withanolides, sitoindosides, and related phytochemicals [7,8,9].

Despite increasing interest in the therapeutic potential of Ashwagandha, the molecular mechanisms underlying the effects of its active compounds remain incompletely understood. WA is generally regarded as the most bioavailable and biologically active constituent of *Withania somnifera*, partly due to its unique structural characteristics, including reactive sites associated with nucleophilic interactions and alkylation [1,3,6]. Pharmacokinetic studies further support its relevance, demonstrating rapid absorption following oral administration of Ashwagandha extract in animal models and higher relative bioavailability compared with other withanolides [10]. Additional reports indicate that WA exhibits approximately 1.5-fold higher relative bioavailability compared with other withanolides [11]. These properties suggest that WA may play a central role in mediating the biological and endocrine effects attributed to Ashwagandha.

Evidence regarding the influence of Ashwagandha on thyroid function remains promising but inconclusive. A double-blind, randomized, placebo-controlled trial in patients with subclinical hypothyroidism reported significant improvements in thyroid-stimulating hormone (TSH), T3, and T4 levels following supplementation with 600 mg/day of Ashwagandha for eight weeks [9]. Conversely, isolated case reports have linked Ashwagandha use to thyroid dysfunction, including painless (silent) thyroiditis, raising concerns about potential adverse endocrine effects [7]. Together, these findings suggest that Ashwagandha may influence thyroid regulation through direct or indirect biochemical and immune-mediated mechanisms. The molecular mechanisms underlying Ashwagandha’s effects remain poorly understood. The observed changes in TSH, T3, and T4 levels raise important questions regarding how its bioactive constituents, particularly WA, interact with endocrine pathways. Current evidence is insufficient to determine whether Ashwagandha directly modulates TSH secretion, influences deiodinase enzymes responsible for the conversion of thyroxine (T4) to triiodothyronine (T3), alters thyroid receptor responsiveness, or affects iodine uptake. Furthermore, limited research has examined its effects in individuals with pre-existing thyroid disorders, leaving uncertainty regarding its safety and potential endocrine consequences in susceptible populations. As a result, the biological pathways through which Ashwagandha exerts its thyroid-related effects remain incompletely characterized and warrant deeper mechanistic investigation [8,9].

One possible explanation for these endocrine effects lies in the close relationship between thyroid dysfunction and inflammatory signalling. Autoimmune thyroid disorders, particularly Hashimoto’s thyroiditis, are frequently associated with chronic systemic inflammation, oxidative stress, and dysregulated immune responses that disrupt thyroid hormone metabolism and epithelial homeostasis [12,13]. Because inflammation plays an important role in thyroid physiology and disease progression, compounds with anti-inflammatory and immunomodulatory properties have attracted growing interest as potential regulators of thyroid-related pathways. In this context, WA represents a promising candidate due to its established effects on cellular stress and inflammatory signalling networks. Previous studies suggested that WA can modulate key molecular pathways, including MAPK, NF-κB, STAT3, and NRF2 signalling, which regulate cytokine production, oxidative stress responses, and metabolic gene expression [14,15,16,17]. Given the close interaction between these pathways and thyroid function, WA may indirectly influence thyroid-related molecular processes through the regulation of upstream inflammatory kinases and transcriptional networks.

Despite increasing evidence supporting the biological activity of WA, its potential impact on thyroid-related molecular pathways has not been systematically characterized, and no thyroid-specific WA transcriptomic datasets are currently available. The existing preclinical and clinical data are inconclusive, focusing primarily on changes in circulating hormone indices with limited mechanistic insight. Previous in silico and in vitro investigations of natural compounds have demonstrated the value of computational approaches for identifying modulators of endocrine and inflammatory targets [2,14]. To our knowledge, no systematic docking or network-based analyses have examined Ashwagandha-derived withanolides against thyroid-related enzymes and upstream kinases. Therefore, the present study employs a cross-tissue, exploratory computational approach integrating WA-responsive transcriptomic data from a non-thyroid epithelial model with curated hypothyroidism-associated genes. The goal is to generate preliminary hypotheses regarding potential regulatory candidates and upstream kinases that can be prioritized for future validation in thyroid-derived experimental systems.

## 2. Results

### 2.1. Transcriptomic Data Acquisition and Differential Gene Expression Analysis

**Figure 1:**
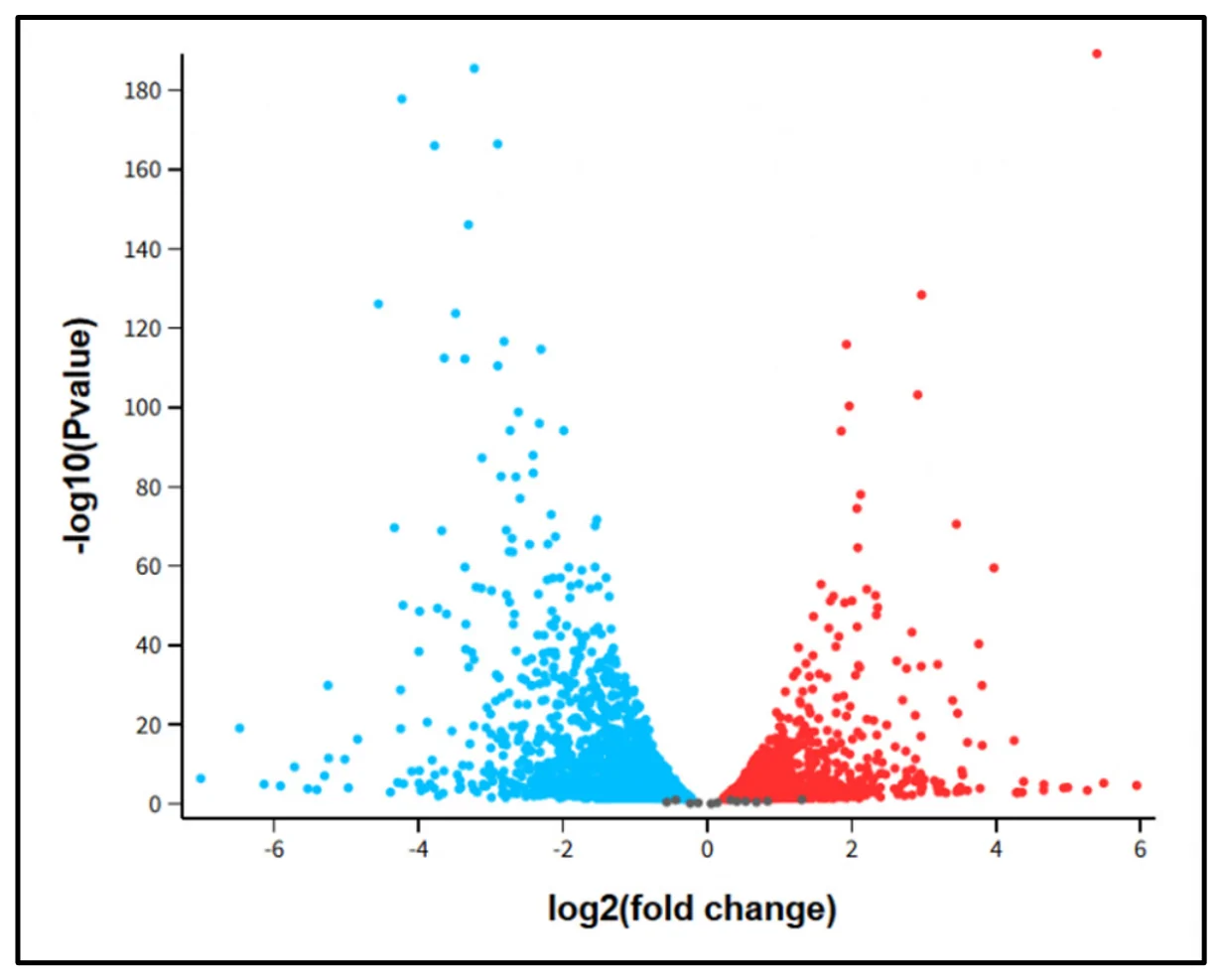
Volcano plot representing the differentially expressed genes in MCF-10A human mammary epithelial cells treated with Withaferin A (WA) compared with vehicle-treated cells. Red dots represent upregulated genes, blue dots represent downregulated genes and gray dots represent genes that did not meet the significance thresholds.

The transcriptomic analysis was first performed using the GSE158085 dataset to characterize gene expression changes following WA treatment. Differential expression analysis of the dataset identified 16,294 genes in the comparison between WA-treated and control mammary epithelial cells. Among these, 7902 genes showed positive log_2_ fold changes, whereas 8392 genes showed negative log_2_ fold changes. Applying the predefined significance thresholds (|log_2_ fold change| > 1 and adjusted *p* < 0.05) resulted in the identification of 1458 significantly differentially expressed genes (DEGs) in WA-treated cells relative to controls. The distribution and magnitude of gene expression changes are illustrated in the volcano plot (Figure 1).

The significantly differentially expressed genes were then intersected with hypothyroidism-associated genes retrieved from the Open Targets Platform using Venny 2.1. The curated Open Targets Platform query for hypothyroidism-related genes returned 80 genes, including classical thyroid regulators such as *TSHR*, *TG*, and *SLC5A5*. Overlap analysis with WA-responsive genes identified 10 overlapping genes (Supplementary Figure S1), namely *SH2D2A*, *C1QTNF6*, *NKX2-5*, *CD226*, *GLIS3*, *DUOX2*, *PAX8*, *STAT4*, *DUOXA2*, and *PTPN22* (Supplementary Table S1).

### 2.2. Functional, Pathway, and Kinase Enrichment Analyses of Target Genes

A protein–protein interaction (PPI) network constructed from the 10 overlapping genes using a STRING confidence threshold of 0.4 contained 10 nodes and 9 edges, with an average node degree of 1.8 and an average local clustering coefficient of 0.75. The network showed significant PPI enrichment (*p* = 1.36 × 10^−10^), indicating more interactions among these proteins than expected for a random set of proteins of similar size. SH2D2A remained an isolated node with no identified interactions at this threshold.

Topological analysis using CytoHubba showed that *PAX8* had the highest degree and MCC values (4/4), followed by *PTPN22* (3/3), while *STAT4*, *DUOX2*, *DUOXA2*, and *CD226* showed degree/MCC values of 2/2 (Supplementary Table S2). Given the small network size, these genes were designated as high-centrality candidate genes.

Sensitivity analysis using a higher STRING confidence threshold of 0.7 substantially reduced network connectivity, retaining only four connected nodes and two interactions: *PTPN22*–*STAT4* and *DUOXA2*–*DUOX2*. All four nodes had identical degree and MCC values of 1. Thus, the centrality pattern observed at the 0.4 threshold was not retained at the higher confidence threshold.

**Figure 2:**
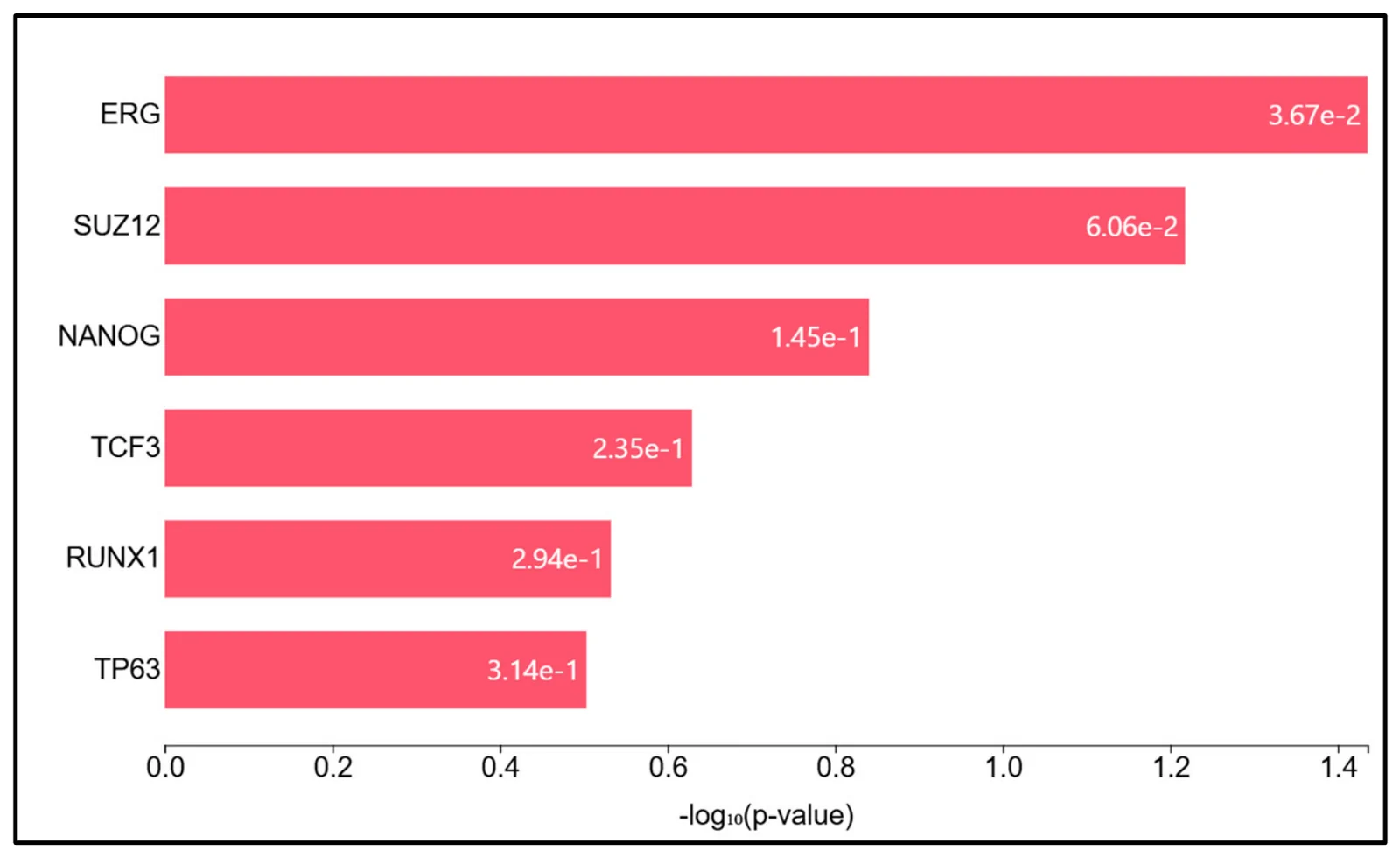
Transcription factor (TF) enrichment analysis of the four selected genes. The *y*-axis represents enriched transcription factors, whereas the *x*-axis represents enrichment significance expressed as −log10(*p*-value). Values displayed within the bars indicate the corresponding *p*-values.

To investigate potential upstream regulators of the four selected genes (*PTPN22*, *STAT4*, *DUOX2*, and *DUOXA2*), transcription factor enrichment analysis was performed using the X2K platform. Six transcription factors were identified as candidate regulators: ERG, SUZ12, NANOG, TCF3, RUNX1, and TP63 (Figure 2). The corresponding raw hypergeometric and BH-adjusted *p*-values are provided in Supplementary Table S3. ERG showed the lowest enrichment *p*-value (3.67 × 10^−2^), followed by SUZ12 (6.06 × 10^−2^), while the remaining predicted TFs showed higher *p*-values. Only ERG had an enrichment *p*-value below 0.05.

To identify potential upstream regulators of the predicted transcription factors, kinase enrichment analysis was performed. The predicted TFs were subjected to PPI network expansion within the X2K workflow, followed by kinase enrichment analysis to identify candidate upstream kinases associated with the expanded regulatory network.

**Figure 3:**
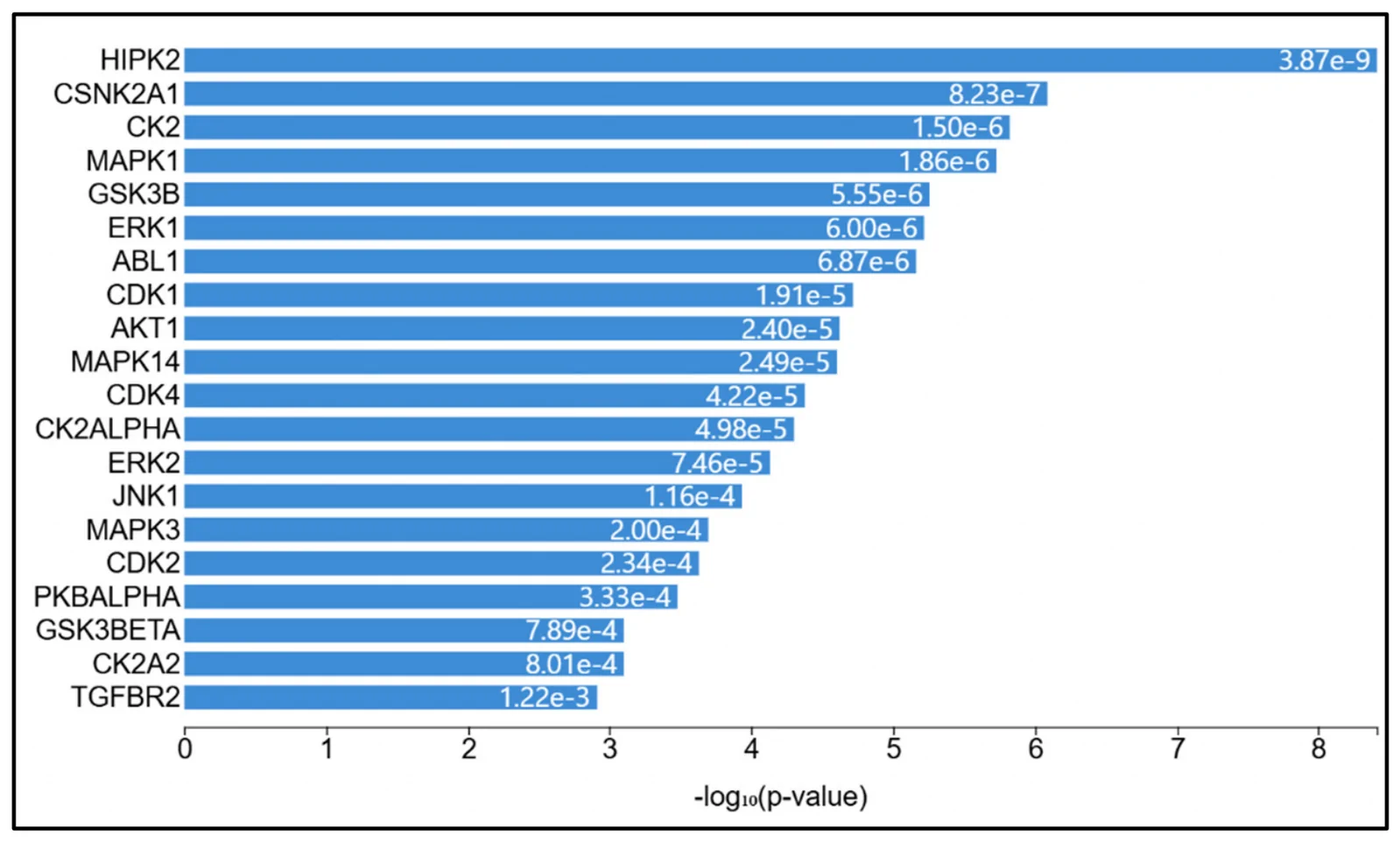
Kinase enrichment analysis of the selected candidate genes. The *y*-axis shows the predicted upstream kinases associated with the selected genes, and the *x*-axis shows enrichment significance as −log10(*p*-value). Values within the bars represent the corresponding *p*-values.

Kinase enrichment analysis (Figure 3) identified several predicted upstream kinases with low enrichment *p*-values, indicating statistical enrichment within the X2K regulatory network. The corresponding raw hypergeometric and BH-adjusted *p*-values for the top 20 kinases are provided in Supplementary Table S4. The top-ranked kinase was HIPK2 (*p* = 1.87 × 10^−9^), followed by GSK3B (8.64 × 10^−8^), CK2ALPHA (1.80 × 10^−7^), CSNK2A1 (2.32 × 10^−7^), and MAPK1 (5.22 × 10^−7^). Other predicted kinases included CDK1, MAPK14, ERK1, CK2, CDK2, CDK4, MAPK3, AKT1, JNK1, ERK2, ABL1, TGFBR2, MAPK8, GSK3BETA, and ATM. The enrichment plot ranked the predicted kinases according to statistical significance, with *p*-values ranging from approximately 1.87 × 10^−9^ to 2.78 × 10^−4^.

**Figure 4:**
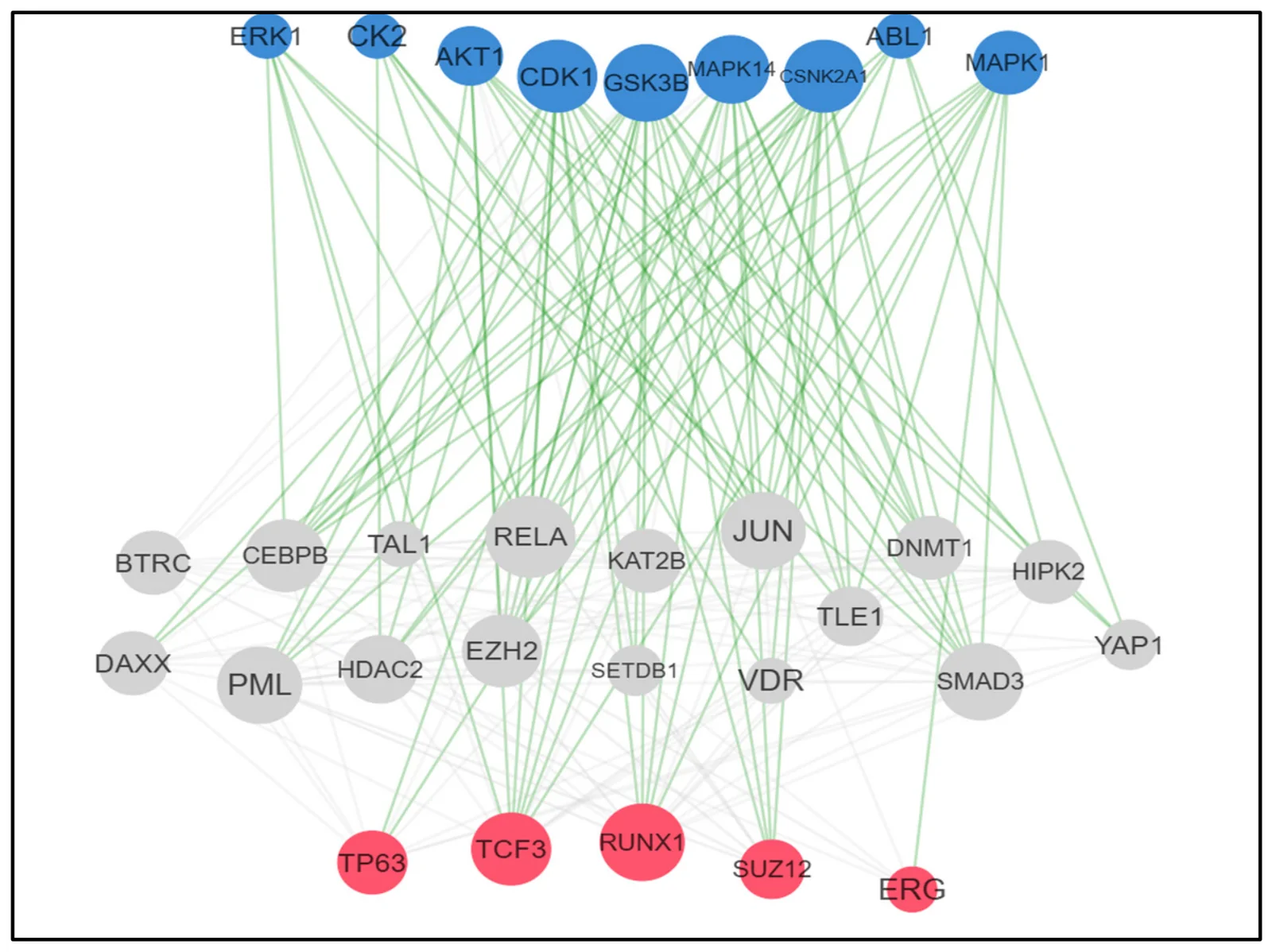
Predicted kinase–transcription factor interaction network. Network visualization of the predicted interactions among candidate upstream kinases (blue), transcription factors (red), and intermediary signalling proteins (grey). Green edges indicate phosphorylation interactions, whereas white edges represent protein–protein interactions (PPIs).

To further examine the potential upstream regulatory relationships associated with the identified transcription factors, a kinase–transcription factor interaction network was constructed. The predicted transcription factors (ERG, SUZ12, NANOG, TCF3, RUNX1, and TP63) were analysed within the X2K workflow to identify candidate upstream kinases and intermediary proteins potentially associated with their regulation. As illustrated in Figure 4, the resulting network comprised three major node types: protein kinases (blue), transcription factors (red), and intermediary signalling proteins (grey). Interactions included phosphorylation (green edges) and protein–protein interactions (white edges). The network included several predicted upstream kinases identified by the enrichment analysis, together with intermediary proteins connecting the kinase and TF layers.

For subsequent molecular docking, enriched kinases were prioritized using predefined criteria that considered enrichment evidence, availability of suitable experimentally resolved protein structures, and literature-supported biological relevance to WA-associated signalling. Based on these criteria, AKT1, MAPK14 (p38α), and MAPK8 (JNK1), ranked 9th, 10th, and 14th in the enrichment analysis, respectively, were selected for further structural investigation. Their enrichment statistics and biological rationale for prioritization are summarized in Table 1. Accordingly, these three kinases were prioritized for molecular docking based on the combined enrichment, structural, and biological criteria.

### 2.3. Molecular Docking Analysis

**Figure 5:**
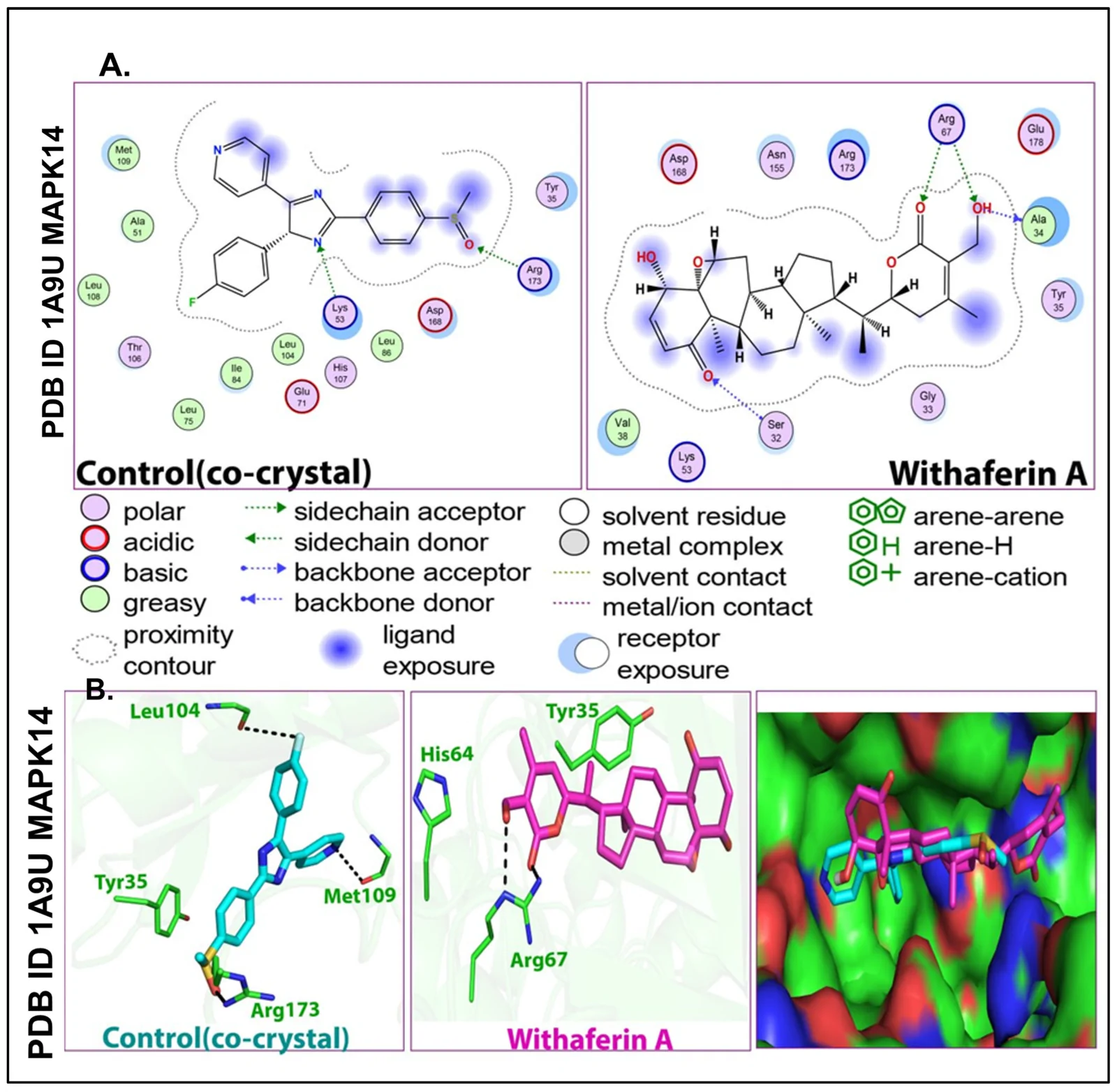
The binding interactions of the co-crystallized inhibitor (SB2) and Withaferin A in the MAPK14 active site (PDB ID: 1A9U): (**A**) Two-dimensional interaction diagrams highlighting hydrogen bonding, hydrophobic interactions, and ligand/receptor exposure. (**B**) Three-dimensional binding poses of the control co-crystal and Withaferin A within the MAPK14 pocket.

Molecular docking was subsequently performed to evaluate the potential direct interaction of WA with these key regulatory kinases. The potential binding interaction between WA and MAPK14 was first evaluated. The crystal structure of p38 MAPK (PDB ID: 1A9U) was used as the structural model, and docking validation was performed against its co-crystallized ligand, SB203580 (SB2) [29]. The control ligand exhibited a well-defined binding pattern within the catalytic pocket, forming key hydrogen bond interactions with Lys53 and Arg173, alongside electrostatic contacts with Asp168 (Figure 5). Hydrophobic interactions were also observed with residues such as Leu75, Ile84, Leu104, and Leu108, which further stabilized the ligand within the pocket. In contrast, WA adopted a distinct binding orientation characterized by an extended conformation occupying a broader region of the binding site. The ligand formed multiple hydrogen bonds, including interactions with Arg67, Ala34, and Ser32. Notably, a bidentate hydrogen-bonding interaction was observed with Arg67. Several of these interactions involved backbone atoms or residues located at the periphery of the binding pocket. The binding mode of WA also included hydrophobic contacts and van der Waals interactions, particularly with residues such as Val38, Tyr35, and Gly33. The docking score of WA was calculated to be −8.9 kcal/mol, compared with −9.0 kcal/mol for the co-crystallized control ligand. Structural superposition further demonstrated that WA partially overlaps with the binding region of the control ligand but extends toward adjacent sub-pockets, showing a distinct binding orientation (Figure 5).

**Figure 6:**
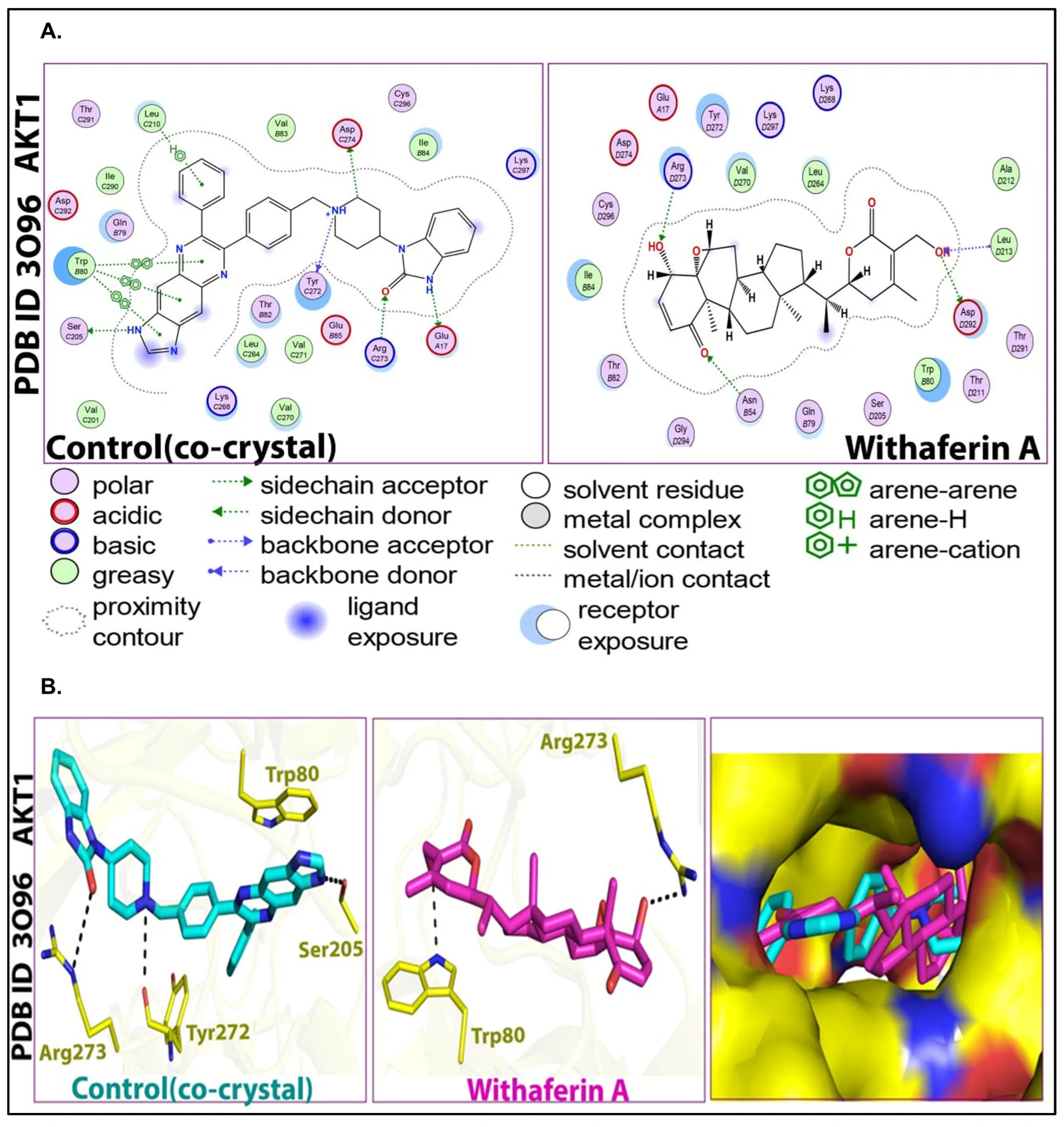
Interaction profiles and binding poses of the control co-crystal (IQO) and Withaferin A in the AKT1 active site (PDB ID: 3O96): (**A**) Two-dimensional protein–ligand interaction maps. (**B**) Three-dimensional docking poses and pocket surface views for the control co-crystal and WA.

Molecular docking analysis was then performed to evaluate the binding of WA to the allosteric site of AKT1 (PDB ID: 3O96), using the co-crystallized allosteric ligand (IQO) as a reference [20]. As shown in Figure 6, the control ligand exhibited a compact binding mode within the allosteric pocket at the PH–kinase domain interface, forming key hydrogen bonds with Ser205, Tyr272, and Arg273, along with stabilizing interactions involving Trp80, and showed an AutoDock Vina docking score of −14.4 kcal/mol. In contrast, WA displayed a docking score of −11.7 kcal/mol and adopted a broader orientation due to its steroidal scaffold, forming hydrogen bonds with Arg273 and Asp292 and contacts with Leu213. However, these interactions were more peripheral and less directional. Structural superposition revealed partial overlap with the control ligand, while WA extended into adjacent sub-pockets. Its binding was predominantly driven by hydrophobic and van der Waals interactions with residues such as Val270, Leu264, Ile84, and Trp80. Overall, although the control ligand exhibited a more favourable docking score and a more focused interaction pattern, WA showed a distinct interaction pattern within the AKT1 allosteric pocket, indicating its potential accommodation at this site.

**Figure 7:**
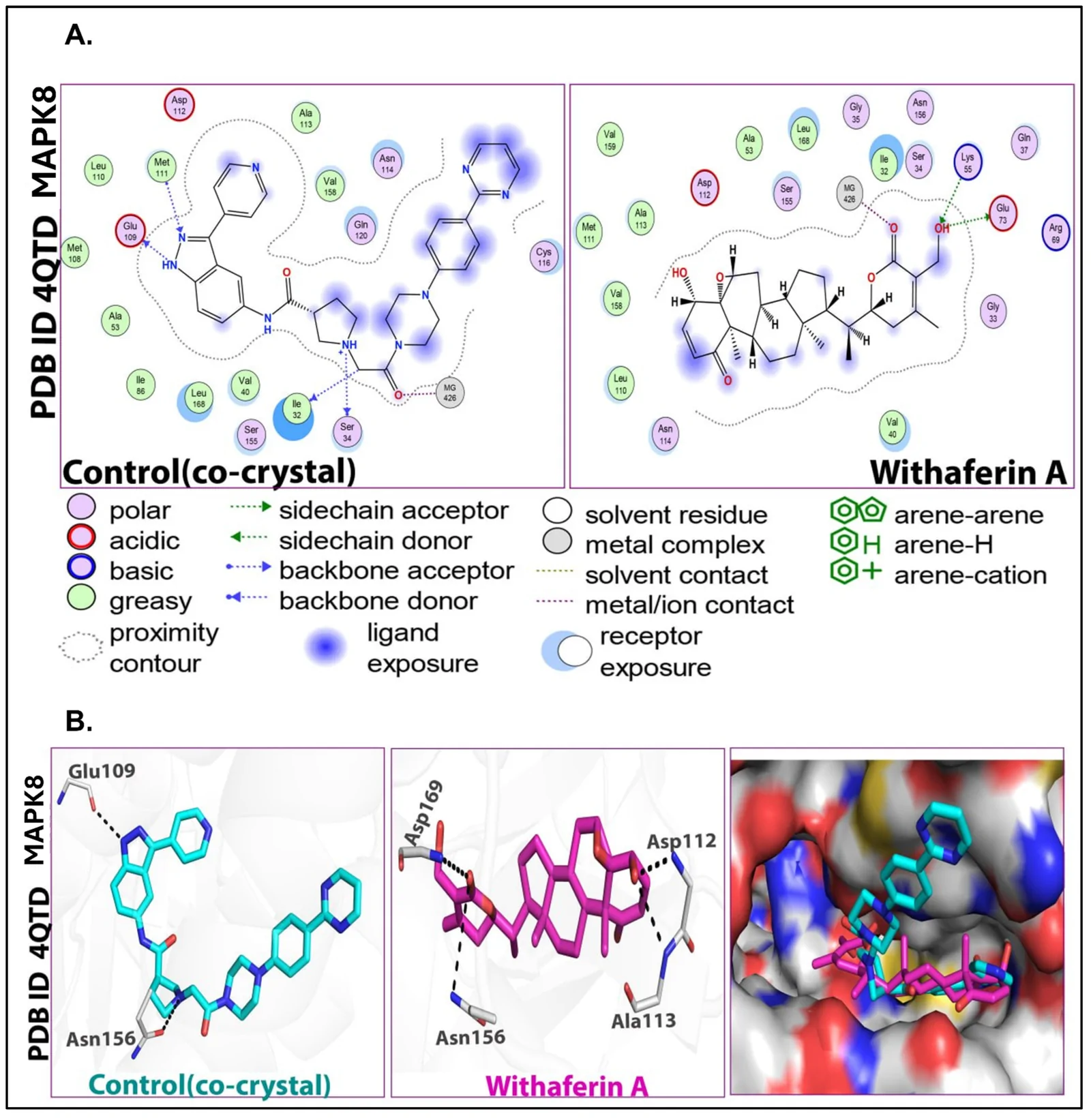
Binding interaction analysis of the JNK1 (MAPK8) complex (PDB ID: 4QTD) with the co-crystallized inhibitor SCH772984 (ligand ID: 38z) and Withaferin A: (**A**) Two-dimensional interaction diagrams of MAPK8 showing binding of the co-crystallized ligand and Withaferin A within the active site. (**B**) Three-dimensional binding conformations and surface representation illustrating the overlap of both ligands within the ATP-binding pocket.

The docking analysis of Withaferin A against JNK1 (MAPK8, PDB ID: 4QTD) showed a predicted binding pose within the ATP-binding pocket. As shown in Figure 7, the co-crystallized ligand (control) formed key hydrogen bonds with Glu109 and Asn156, whereas WA formed hydrogen bonds with Asp112, Asp169, and Asn156. Notably, both ligands interacted with Asn156, representing a common interaction feature. WA additionally interacted with Asp112 and Asp169, indicating a distinct interaction pattern within the binding pocket. The 3D visualization further showed partial overlap and differences in binding orientation between the two ligands. The co-crystallized ligand showed a more favourable docking score (−10.9 kcal/mol) than WA (−9.2 kcal/mol). Overall, WA adopted a predicted binding pose within the JNK1 ATP-binding pocket, with an interaction pattern distinct from that of the control ligand.

### 2.4. Dynamic Simulation Analysis

#### 2.4.1. MD Simulation for MAPK14

**Figure 8:**
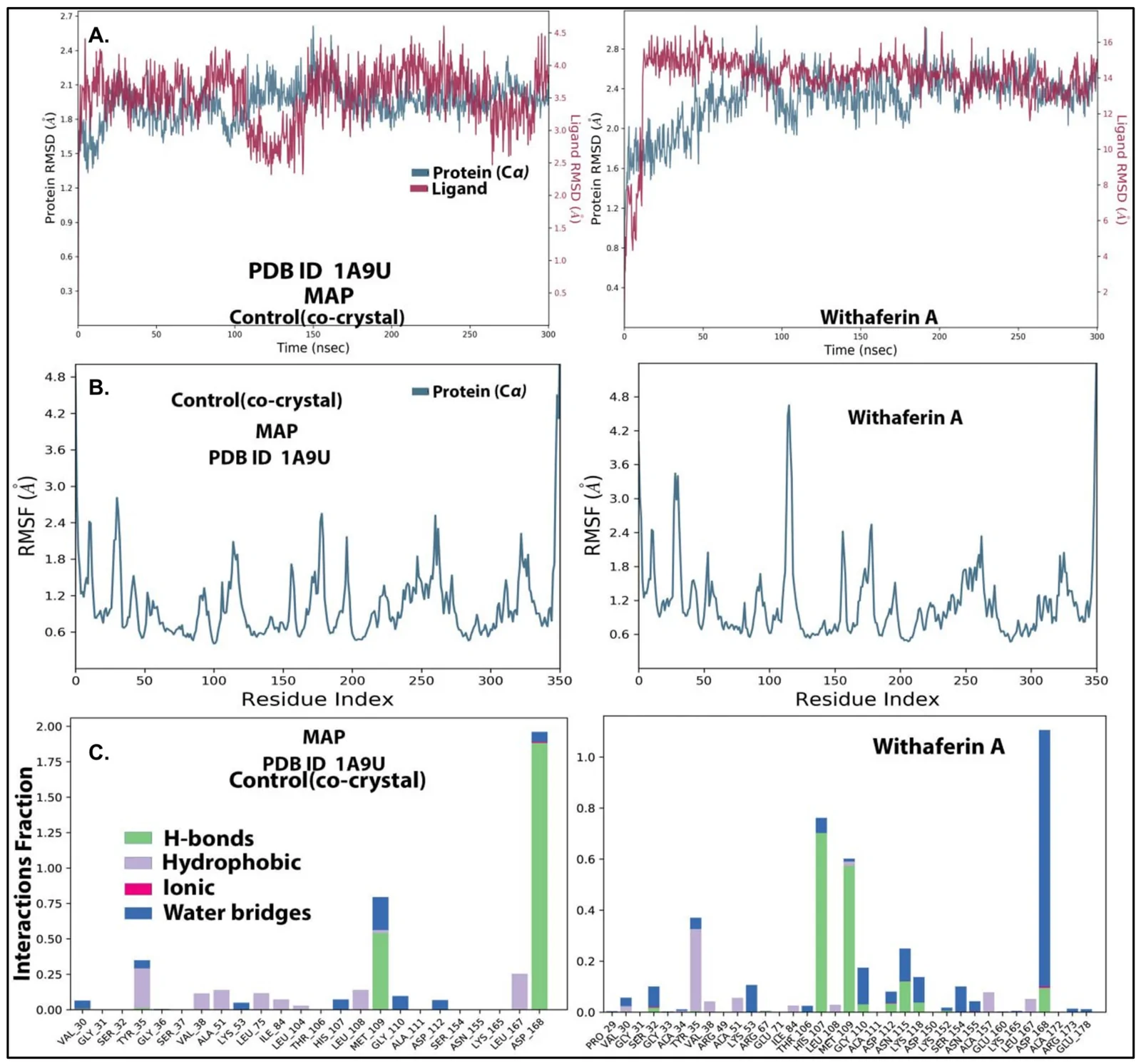
Molecular dynamics simulation analysis of the complex structure of MAP kinase 14 with the control inhibitor [4-[5-(4-fluorophenyl)-2-(4-methanesulfinyl-phenyl)-3H-imidazol-4-yl]-pyridine] and WA over a 300 ns simulation: (**A**) root mean square deviation (RMSD); (**B**) root mean square fluctuation (RMSF); (**C**) protein–ligand contacts.

The stability of the MAP kinase 14–ligand complexes was evaluated using root mean square deviation (RMSD) analysis during a 300 ns molecular dynamics simulation (Figure 8). In the control co-crystal complex, the protein backbone RMSD fluctuated between approximately 1.7 and 2.2 Å, indicating preservation of protein structural integrity throughout the simulation. The ligand RMSD remained within 3–4.5 Å, with a transient reduction observed at approximately 110–140 ns. In contrast, the WA complex showed an initial increase in protein RMSD to approximately 2.5 Å during the first 50–80 ns, followed by relatively stable values. However, the ligand RMSD fluctuated markedly between 12 and 16 Å, showing substantial deviation from the initial docking pose. Overall, the protein backbone RMSD remained below approximately 3 Å in both systems, whereas the WA ligand showed considerably greater RMSD fluctuations than the control ligand.

Root mean square fluctuation (RMSF) analysis showed that most residues remained below 2 Å in both complexes. In the control complex, increased flexibility was observed mainly at residues 20–40, 170–200, and 250–270, while the C-terminal region (~350) displayed the highest fluctuation (~4.5 Å), consistent with terminal flexibility. Similarly, the WA complex showed localized increases in flexibility, particularly at residues 25–35 and 110–120, with the latter reaching approximately 4.6 Å. Outside these localized regions, relatively low residue fluctuations were observed across both complexes.

Analysis of protein–ligand interactions indicated that the control ligand formed hydrogen bonds, particularly with ASP168 and MET109, along with hydrophobic and water-mediated contacts that aid in stabilizing the ligand. WA formed hydrogen bonds with HIE107 and MET109, together with hydrophobic and water-mediated interactions, including a water-mediated contact with ASP168.

#### 2.4.2. MD Simulation for AKT1

**Figure 9:**
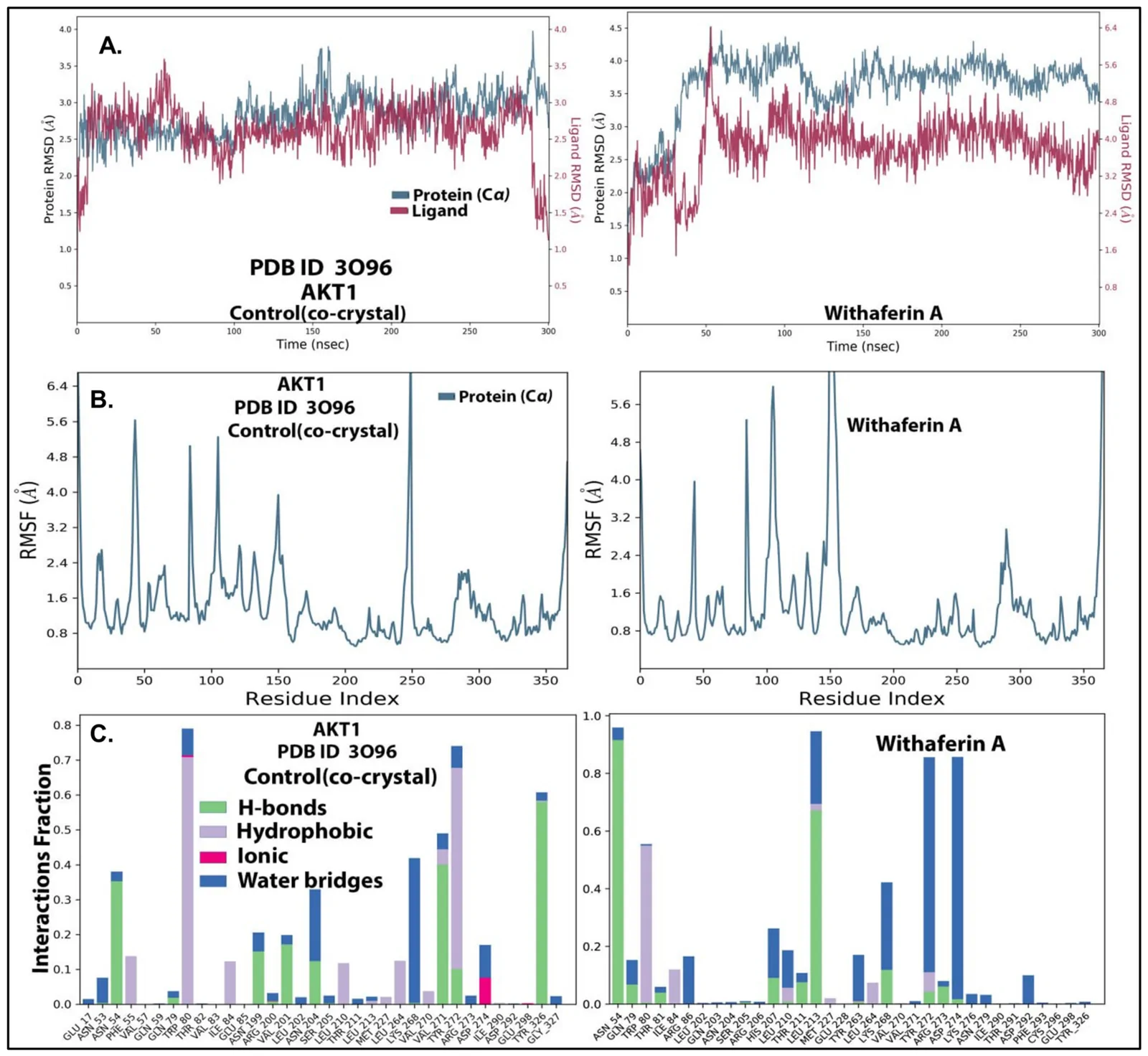
Molecular dynamics simulation of the AKT1–IQO and AKT1–Withaferin A complexes over a 300 ns simulation: (**A**) root mean square deviation (RMSD); (**B**) root mean square fluctuation (RMSF); (**C**) protein–ligand profiles of both complexes.

The structural stability of the AKT1–ligand complexes (PDB ID: 3O96) was assessed through 300 ns molecular dynamics simulations using RMSD, RMSF, and protein–ligand interaction analyses (Figure 9). In the control co-crystal complex, the protein backbone RMSD fluctuated between approximately 2.2 and 3.3 Å, indicating maintenance of structural integrity. The co-crystal ligand exhibited RMSD values mainly within 2.2–3.2 Å, with relatively limited fluctuations around its initial configuration. In comparison, the WA-bound complex displayed a rapid rise in protein RMSD during the initial 30–50 ns, reaching approximately 3.5–4.2 Å, followed by relatively consistent values for the remainder of the simulation, suggesting an early conformational adaptation of AKT1 upon ligand binding. Ligand RMSD values fluctuated between approximately 3.0 and 5.0 Å, showing moderately greater mobility than the control ligand.

The RMSF analysis revealed that most residues in both systems exhibited fluctuations below 2 Å. In the control complex, elevated flexibility was observed at several localized regions, particularly around residues 40–60, 90–120, 140–160, and near residue 250, where peaks reached approximately 5–6 Å, likely corresponding to flexible loop regions. Likewise, the WA complex showed increased fluctuations at residues approximately 40–60, 90–150, and near 280–300, with maximum values approaching 6 Å. These higher fluctuations were localized to specific protein regions.

Protein–ligand interaction profiling demonstrated that the control ligand formed multiple interactions, including hydrogen bonding, hydrophobic interactions, ionic contacts, and water bridges, particularly with ARG273, TYR272, ASN279, and GLY211. In contrast, WA showed hydrogen-bond and water-mediated interactions involving ASN54, LEU210, ARG273, and TYR272.

#### 2.4.3. MD Simulation for JNK1

**Figure 10:**
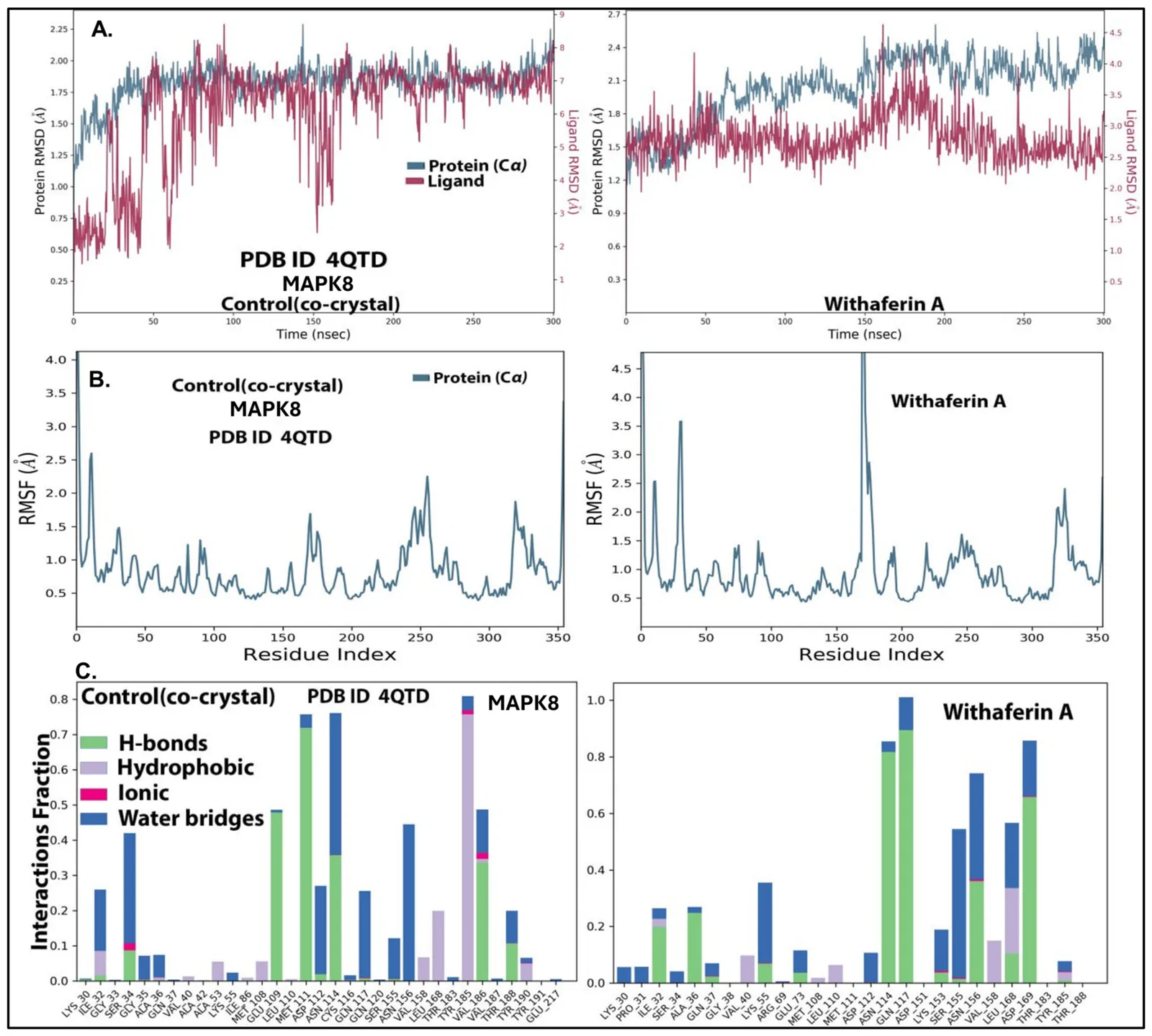
Molecular dynamics simulation of the complex structure of MAPK8 with the control inhibitor SCH772984 (ligand ID: 38z) and Withaferin A: (**A**) root mean square deviation (RMSD); (**B**) root mean square fluctuation (RMSF); (**C**) protein–ligand interaction fractions showing binding type.

The structural behaviour of the JNK1 (MAPK8)–ligand complexes (PDB ID: 4QTD) was assessed using 300 ns molecular dynamics simulations through RMSD, RMSF, and protein–ligand interaction analyses (Figure 10). In the control co-crystal complex, the protein backbone RMSD fluctuated between approximately 1.5 and 2.1 Å, indicating preservation of structural integrity throughout the simulation. The co-crystal ligand exhibited RMSD values primarily within 6–8 Å, with transient fluctuations during the early stage of the simulation, suggesting conformational adaptation before stabilization within the binding pocket. In contrast, the Withaferin A complex displayed a gradual increase in protein RMSD during the first 50–80 ns, reaching approximately 2.0–2.5 Å, followed by relatively consistent values for the remainder of the simulation. Ligand RMSD values fluctuated mainly between 2.5 and 3.5 Å, lower than those observed for the control ligand.

The RMSF analysis showed that most residues in both complexes exhibited fluctuations below 2 Å. In the control complex, elevated flexibility was observed near residues 10–30, 160–180, 240–270, and 310–330, where peaks approached approximately 2–2.5 Å, consistent with localized loop mobility. Similarly, the WA-bound system showed increased fluctuations at residues 20–40 and particularly around 170–180, where RMSF values reached approximately 4.5 Å. Outside these localized regions, relatively low residue fluctuations were observed throughout both systems.

Protein–ligand interaction analysis revealed that the control ligand established multiple interactions, including hydrogen bonds and hydrophobic contacts, particularly involving MET111, ASP169, and TYR185, together with water-mediated interactions. In comparison, WA showed hydrogen-bond, hydrophobic, and water-mediated interactions involving ASN114, GLN117, VAL158, and ASP169.

## 3. Discussion

In this study, we sought to explore potential molecular relationships through which Withaferin A (WA), a bioactive compound of Ashwagandha (*Withania somnifera*), may be relevant to thyroid-associated processes. Although this medicinal plant has long been associated with endocrine regulation and metabolic balance in traditional medicine, the molecular pathways linking its bioactive compounds to thyroid-related processes remain insufficiently understood [8]. Therefore, we employed a cross-tissue, exploratory computational approach integrating WA-responsive RNA-seq data from GSE158085 with curated hypothyroidism-associated genes. The analysis aimed to identify overlapping genes and potential regulatory candidates for further computational investigation. This integration identified a set of overlapping genes (PTPN22, STAT4, DUOX2, and DUOXA2) that provided the basis for examining their potential relevance to thyroid-associated biological processes. These genes have established roles in thyroid function and disease. DUOX2 and DUOXA2 are components of the H_2_O_2_-generating system required for thyroid hormone biosynthesis [30,31,32], while PTPN22 and STAT4 are immune-regulatory genes whose variants have been associated with autoimmune thyroid diseases, including Hashimoto’s thyroiditis [33,34]. Thus, the overlap includes WA-responsive genes with previously reported roles in thyroid hormone biosynthesis and immune-regulatory processes. However, because these transcriptional changes were identified in a non-thyroid cellular model, their relevance to thyroid cells remains hypothetical and requires validation in thyroid-derived experimental systems.

The TF enrichment analysis further identified ERG, SUZ12, NANOG, TCF3, RUNX1, and TP63 as predicted candidate regulators (Figure 2). Among these, ERG showed the strongest enrichment, with a raw *p*-value below 0.05. Together, these findings suggest potential regulatory relationships between the selected genes and predicted TFs. Several of the predicted transcription factors are associated with kinase-dependent signalling pathways, and their activity is often controlled by phosphorylation events mediated by signalling kinases [25,35,36]. Previous studies have demonstrated that withanolides, particularly WA, can modulate kinase signalling pathways. In this context, the candidate upstream kinases identified in our analysis may be relevant to cellular stress, inflammatory, and metabolic signalling pathways [37,38]. Their identification provides a basis for further investigation of potential WA-associated regulatory relationships. These findings provide biological context for the predicted kinase associations identified in our analysis; however, these associations are computational predictions rather than evidence of direct kinase activity or a WA-mediated regulatory mechanism and therefore warrant further experimental investigation.

MAPK14 (p38α) is a key member of the p38 MAP kinase family involved in inflammatory and stress-response signalling pathways [39,40,41]. Its identification in the kinase enrichment analysis, along with evidence linking p38 signalling to cellular responses to WA, supported its selection for structural investigation. Docking analysis showed that WA and the control ligand occupied partially overlapping regions of MAPK14, with distinct interaction patterns. While the control ligand formed fewer but more specific and deeply buried interactions, WA showed multiple hydrogen bonds and extensive hydrophobic interactions, with several contacts located toward the periphery of the binding pocket (Figure 5). Despite comparable docking scores (−8.9 and −9.0 kcal/mol for WA and the control, respectively), their distinct orientations and interaction patterns suggest different predicted binding modes rather than equivalent binding affinity or kinase inhibition. Beyond its established roles in inflammatory and stress-response signalling, MAPK14 has been implicated in cancer-associated inflammation and several oncogenic processes [42]. Its potential relevance to thyroid-associated processes is supported by the involvement of p38 MAPK signalling in inflammatory and oxidative stress responses, which have been implicated in autoimmune thyroid disease, as well as in thyroid hormone-related regulatory processes, including deiodinase pathways [12,13,25]. Moreover, p38 MAPK signalling has been linked to thyroid hormone-related regulatory processes, including deiodinase pathways [26]. These observations provide biological context for investigating MAPK14 as a candidate kinase in the present study. Regarding AKT1, this serine/threonine kinase is a key component of the PI3K/AKT pathway regulating cell survival, growth, metabolism, and proliferation [35]. WA has been shown to markedly suppress MMP-9 expression and enzymatic activity in metastatic cancer cells in association with reduced AKT phosphorylation, thereby reducing cancer cell invasion without causing cytotoxicity [21]. These findings support an effect of WA on AKT-related signalling but do not establish direct allosteric inhibition of AKT1. PI3K/AKT signalling has also been implicated in thyroid pathological processes, particularly thyroid cancer and inflammatory signalling [22,43], providing biological context for AKT1 in the present analysis. Consistent with our docking results, computational studies have also reported potential interactions between WA and AKT1 [19]. Together, these findings support AKT1 as a candidate for further investigation of WA-associated signalling. Finally, MAPK8 (JNK1) is a component of the JNK pathway involved in cellular responses to oxidative stress and inflammatory stimuli [13,44]. WA has been reported to modulate JNK signalling, including phosphorylation-dependent stress responses [6,45], providing biological context for the identification of MAPK8 in our analysis. The predicted interaction of WA with the JNK1 binding site further supports its investigation as a candidate kinase, although it does not establish ATP-competitive inhibition or a direct effect on thyroid function.

The MD simulation showed that the MAPK14 protein backbone remained relatively stable in both the control and WA systems throughout the 300 ns trajectory. In contrast, ligand behaviour differed markedly between the two systems. The substantially higher RMSD of WA indicates considerable rearrangement from its initial docking pose, whereas the control ligand remained closer to its initial configuration. The RMSF profiles showed that most residues remained relatively stable, with increased flexibility restricted to specific regions. WA also displayed a broader pattern of hydrogen-bond, hydrophobic, and water-mediated interactions. Overall, these observations indicate substantial mobility of WA within the MAPK14 system and suggest that its initial docked orientation was not maintained throughout the trajectory.

The AKT1 simulation showed a different dynamic profile. WA exhibited moderately greater mobility than the control ligand but substantially less displacement than observed in the MAPK14 system. Most residues showed relatively low fluctuations, with differences confined to localized regions. Importantly, WA maintained several hydrogen-bond and water-mediated contacts, including interactions involving ARG273 and TYR272, which were also observed in the control complex. These observations suggest that WA remained associated with the AKT1 binding region despite greater mobility than the co-crystallized ligand.

A distinct pattern was observed for JNK1, where WA showed lower positional deviation than the control ligand and maintained multiple hydrogen-bond, hydrophobic, and water-mediated contacts, including ASP169. Protein flexibility remained generally low, apart from localized fluctuations around residues 170–180. Together, these observations suggest a comparatively consistent configuration of WA within the JNK1 binding region during the simulated trajectory. Overall, the docking and MD analyses revealed distinct interaction and dynamic profiles of WA across the three candidate kinases, with the greatest ligand displacement observed for MAPK14 and comparatively lower positional deviations for AKT1 and JNK1. These structural predictions support potential interactions between WA and the selected kinases but do not establish binding affinity or functional kinase inhibition. Taken together, the findings of this study identify candidate relationships among WA-responsive genes, predicted transcription factors, and upstream kinases potentially relevant to thyroid-associated molecular processes. Rather than establishing a direct WA–kinase–TF–gene mechanism, the integrated analysis provides testable hypotheses for further investigation using kinase activity assays, gene-expression studies, and thyroid-derived experimental models.

## 4. Materials and Methods

### 4.1. Study Design

This research utilized a comprehensive computational approach to explore molecular pathways related to the thyroid that might be affected by Withaferin A (WA). Transcriptomic data from cells treated with WA, available publicly, were examined to pinpoint genes with altered expression levels. These differentially expressed genes (DEGs) were then cross-referenced with genes linked to hypothyroidism to establish a gene set responsive to WA and relevant to the thyroid. The genes that overlapped were then used to construct a protein–protein interaction (PPI) network, prioritize hub genes, validate disease associations, and conduct enrichment analyses for transcription factors and kinases. This was followed by structure-based molecular docking and molecular dynamics (MD) simulation of selected kinase targets.

### 4.2. Transcriptomic Dataset Selection and Differential Expression Analysis

Differential gene expression analysis was performed using an RNA-sequencing dataset obtained from the Gene Expression Omnibus (GEO) under accession number GSE158085 (https://www.ncbi.nlm.nih.gov/geo/query/acc.cgi?acc=GSE158085; accessed on 4 April 2026). The dataset includes normal mammary epithelial cells (MCF-10A) treated with Withaferin A (WA) or vehicle control (DMSO), with three biological samples per group. Because no thyroid-specific WA-treated transcriptomic dataset was available at the time of analysis, this dataset was used to identify WA-responsive transcriptional changes that could subsequently be filtered for thyroid relevance. The dataset was generated using the Illumina NovaSeq 6000 platform (GPL24676). The original study provided HTSeq-derived gene-level read counts and FPKM values as processed expression data.

Differential expression analysis was performed using the GEO2R web interface. For this RNA-seq dataset, GEO2R uses NCBI-computed gene-level count data as input and applies the DESeq2 framework; therefore, the submitter-provided FPKM values were not used for differential expression testing, and limma-voom was not applied. DESeq2 normalization was used to account for differences in sequencing depth between samples. The experimental design compared WA-treated samples with the corresponding DMSO-treated controls (*n* = 3 per group). Gene identifiers and annotations were retained from the GEO2R/NCBI output associated with the dataset. No additional batch correction was applied because the samples belonged to a single experimental dataset with no reported batch structure. The complete GEO2R results table was downloaded for subsequent analysis and visualization. Genes with |log2FC| > 1 and Benjamini–Hochberg-adjusted *p* < 0.05 were considered statistically significant differentially expressed genes (DEGs). A total of 1459 significant DEGs meeting both criteria were retained for downstream analyses.

### 4.3. Identification of Hypothyroidism-Related WA-Responsive Genes

To refine the disease–gene association analysis, genes specifically linked to hypothyroidism were retrieved using the Open Targets Platform (https://platform.opentargets.org). The search was performed under the Disease module using the term “hypothyroidism” (EFO:0004705). Only genes with a global association score ≤ 0.5 were retained to ensure high-confidence associations. WA-responsive differentially expressed genes (DEGs) identified from the GSE158085 dataset were then intersected with this hypothyroidism-specific gene set using the Venny 2.1 tool to obtain a focused subset of genes potentially relevant to WA’s endocrine-associated effects. This disease-stratified intersection yielded the hypothyroidism-related WA-responsive candidates used for subsequent protein–protein interaction (PPI) network construction, hub-gene prioritization, transcription factor enrichment, and kinase regulatory analysis.

### 4.4. Protein–Protein Interaction Network Construction and Centrality Analysis

The overlapping genes were analysed using the STRING database (version 11.5) (https://string-db.org/) to construct a protein–protein interaction (PPI) network for *Homo sapiens*. A minimum interaction confidence score of 0.4 (medium confidence) was initially applied. The resulting network was imported into Cytoscape (version 3.10.0) for visualization and topological analysis. Network centrality was assessed using the CytoHubba plugin based on degree centrality and Maximal Clique Centrality (MCC). Given the small network size, genes with relatively high centrality values were considered high-centrality candidate genes rather than definitive hub genes. To assess the sensitivity of the centrality pattern to the selected STRING confidence threshold, the analysis was repeated using a stricter confidence score of 0.7 (high confidence), and the resulting network connectivity and centrality patterns were compared with those obtained at the 0.4 threshold.

### 4.5. Identification of Upstream Kinases Potentially Regulating Candidate Gene Expression

To explore the putative upstream regulatory mechanisms controlling the expression of five selected genes (*PTPN22*, *STAT4*, *DUOX2*, *DUOXA2*, and *CD226*), transcription factor enrichment analysis was performed. The selected genes were submitted to the eXpression2Kinases (X2K Web) platform (https://maayanlab.cloud/X2K/) (accessed on 10 April 2026) to predict transcription factors that may regulate their expression based on curated gene regulatory networks and experimental datasets. Transcription factor enrichment was performed using the ChEA & ENCODE Consensus library within X2K. The background organism was set to “Both” (human and mouse), with the enrichment background defined by the target-gene universe represented in the selected ChEA & ENCODE Consensus library. Six predicted transcription factors (*ERG*, *SUZ12*, *NANOG*, *TCF3*, *RUNX1*, and *TP63*) were identified and carried forward to the subsequent network-expansion analysis. The enrichment analysis ranks transcription factors according to their statistical association with the input gene set. Significance was estimated using hypergeometric *p*-values reported by X2K. To account for multiple testing, the raw hypergeometric *p*-values were adjusted using the Benjamini–Hochberg false discovery rate (FDR) method. Complete raw and adjusted *p*-values are provided in Supplementary Table S3. The results were visualized as a bar plot representing −log10(*p*-value) scores to highlight the most relevant predicted candidate regulators.

Kinase enrichment analysis was performed to identify kinases that may regulate the predicted transcription factors. The transcription factors were further analysed through the X2K workflow, including protein–protein interaction network expansion using the Genes2Networks (G2N) module with BioGRID 2017, IntAct 2017, KEGG, MINT 2017, ppid, and Stelzl as interaction resources, followed by kinase enrichment based on known kinase–substrate interactions using the KEA 2018 kinase–substrate interaction. Kinases were ranked according to statistical significance using enrichment *p*-values, and the top enriched kinases were identified as predicted candidate upstream regulators of the transcription factor network and selected gene expression.

### 4.6. Molecular Docking and Dynamic Simulation

Molecular docking was performed to investigate the binding interactions of WA with three kinases prioritized based on enrichment, structural availability, and biological relevance. The three-dimensional crystal structures of MAPK14 (p38; PDB ID: 1A9U), MAPK8 (JNK1; PDB ID: 4QTD), and AKT1 (PDB ID: 3O96) were retrieved from the Protein Data Bank (PDB), while the three-dimensional structure of WA (CID: 265237) was downloaded from the PubChem database in SDF format.

Protein preparation was carried out using AutoDock Tools (ADT, version 1.5.6). All crystallographic water molecules, co-crystallized ligands, and non-essential heteroatoms were removed from the protein structures. The receptor structures were inspected for unresolved residues, loops, and side chains before docking. Unresolved regions outside the defined ligand-binding pockets were not reconstructed, as the docking search spaces were restricted to the experimentally defined ligand-binding regions. Receptors were prepared using the standard ADT workflow, including the addition of polar hydrogen atoms and assignment of Kollman united-atom charges. No separate pKa-based protonation-state calculation was performed. WA was energy-minimized, Gasteiger charges were assigned, and the structure was converted to PDBQT format. WA was prepared in its neutral form for docking, and alternative protonation or tautomeric states were not explicitly sampled.

Docking simulations were performed using AutoDock Vina implemented in PyRx (version 0.8). The docking grid was centred on the native ligand-binding pocket of each kinase. For MAPK14 (1A9U), the search space was defined around the crystallographic SB203580 (SB2)-binding pocket; for AKT1 (3O96), it was defined around the crystallographic IQO-binding allosteric pocket at the PH–kinase domain interface; and for MAPK8/JNK1 (4QTD), it was defined around the crystallographic SCH772984 (38Z)-binding pocket. For each structure, the docking box was adjusted to encompass the corresponding co-crystallized ligand and surrounding binding-site residues. Vina exhaustiveness was set to 8, and 10 binding modes were generated per docking run. The conformation with the lowest AutoDock Vina docking score was selected for subsequent analyses.

Protein–ligand interactions, including conventional hydrogen bonds, hydrophobic interactions, π-interactions, van der Waals contacts, and electrostatic interactions, were analysed using Discovery Studio Visualizer (BIOVIA, Dassault Systèmes, San Diego, CA, USA). Two-dimensional interaction diagrams were generated to identify interacting residues and characterize the binding patterns of the native ligand and WA. Three-dimensional binding conformations and ligand superimposition were analysed using PyMOL (version 2.5) to assess the binding orientation and accommodation of WA within the corresponding ligand-binding pocket. AutoDock Vina docking scores were interpreted as predicted binding affinities (kcal/mol) and used as relative ranking metrics rather than experimentally measured thermodynamic binding energies.

The stability of the docked protein–ligand complexes was further evaluated by molecular dynamics simulation using the Desmond Molecular Dynamics System (Schrödinger LLC, New York, NY, USA) with the OPLS4 force field. Each complex was solvated in an orthorhombic box containing TIP3P water molecules and neutralized with appropriate counterions. After energy minimization and equilibration under NVT and NPT ensembles at 300 K and 1 atm, a 300 ns production simulation was performed under periodic boundary conditions. Trajectory analyses, including root mean square deviation (RMSD), root mean square fluctuation (RMSF), and protein–ligand interaction profiles (hydrogen bonds, hydrophobic interactions, ionic interactions, and water bridges), were carried out using the Simulation Interaction Diagram (SID) module in Desmond. A single 300 ns production trajectory was generated for each complex; therefore, the simulations were interpreted as providing initial dynamic insights rather than replicate-based estimates of comparative binding stability.

## 5. Conclusions

This study presents an integrated computational framework to explore potential associations between WA-responsive transcriptional changes and thyroid-related genes. By combining transcriptomic analysis, network analysis, regulatory enrichment, molecular docking, and molecular dynamics simulations, candidate genes and upstream regulators were identified for further investigation in a thyroid context. Regulatory enrichment prioritized MAPK14, MAPK8, and AKT1 as candidate upstream kinases, whose regulatory activity does not necessarily require differential expression of their encoding genes.

Docking and MD simulations provided structural hypotheses for potential interactions between WA and these kinases, with target-specific dynamic behaviour rather than uniformly stable binding across the investigated systems. Notably, the substantial mobility of WA in the MAPK14 complex indicates that its initial docked orientation was not maintained throughout the simulation. Overall, the findings provide an exploratory basis for prioritizing candidate regulatory relationships and WA–kinase interactions for further investigation. Their biological relevance and potential involvement in thyroid-related processes require validation in thyroid-derived experimental models.

## 6. Limitations

Several limitations should be considered when interpreting these findings. First, the transcriptomic analysis was based on WA-treated MCF-10A mammary epithelial cells because no suitable WA-treated thyroid transcriptomic dataset was publicly available. Consequently, associations with thyroid-related genes cannot be assumed to represent transcriptional responses in thyroid cells, as tissue-specific differences in gene regulation and signalling may substantially alter the response to WA. The transcriptomic component should therefore be regarded as a cross-tissue, hypothesis-generating analysis.

Second, the successive DEG–TF–kinase analyses introduce uncertainty at each level of computational inference. The predicted kinases represent potential upstream regulators and need not themselves be differentially expressed, as kinase activity can also be regulated post-translationally. These regulatory relationships therefore represent predictions rather than established mechanisms.

Finally, docking and MD simulations provide exploratory predictions and do not establish kinase inhibition. The docking protocol lacked re-docking/RMSD validation, and conventional non-covalent docking may not fully capture the electrophilic nature and potential covalent interactions of WA. MD simulations were limited to a single 300-ns trajectory per complex. Further experimental and computational validation is required.

## Figures and Tables

**Table 1:** Enrichment statistics and biological rationale for kinase prioritization in molecular docking.

Kinase	Enrichment Rank	*p*-Value	PDB ID	Biological Rationale
AKT1	9	2.40 × 10^−5^	3O96	WA-modulated AKT signalling and relevance to thyroid signalling [18,19,20,21,22]
MAPK14 (p38α)	10	2.49 × 10^−5^	1A9U	WA-responsive p38 MAPK signalling and inflammation/stress relevance [23,24,25,26]
MAPK8 (JNK1)	14	1.16 × 10^−4^	4QTD	WA-responsive JNK signalling and stress-response relevance [24,27,28]

## Data Availability

The datasets generated during and/or analysed during the current study are available from the corresponding author upon reasonable request.

## Supplementary Materials

The following supporting information can be downloaded at https://www.mdpi.com/article/10.3390/ijms27198926/s1.
