# Supplementary material for: Integrated Transcriptomic and Network Analysis Identifies Candidate Kinase-Mediated Thyroid-Associated Targets of Withaferin A
Source: Int J Mol Sci. 2026 Oct 8;27(19):8926. doi: 10.3390/ijms27198926 (PMC13665287; doi:10.3390/ijms27198926)
Supplement: Supplementary file 1 [file ijms-27-08926-s001.zip › ijms-4546509-supplementary.pdf]

Supplementary File

Integrated Transcriptomic and Network Analysis Identifies Candidate Kinase-Mediated Thyroid-Associated Targets of Withaferin A

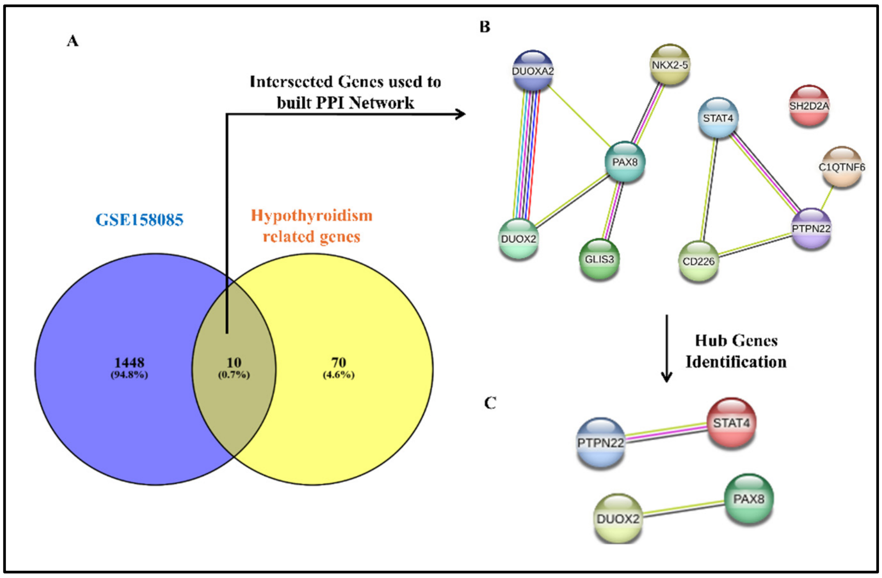

Figure S1: Identification and network analysis of overlapping WA-responsive and hypothyroidism-associated genes. (A) Venn diagram showing the overlap between differentially expressed genes (DEGs) from the GSE158085 dataset and hypothyroidism-associated genes obtained from the Open Targets Platform. Ten overlapping genes were identified. **(B)** STRING protein–protein interaction (PPI) network of the 10 overlapping genes. Edge thickness reflects the confidence score of protein–protein associations. **(C)** High-centrality candidate gene subnetwork identified using CytoHubba in Cytoscape, showing PTPN22, STAT4, DUOX2, and PAX8 and their associated interactions.

Table S1. Differential expression of WA-responsive genes overlapping with hypothyroidism-associated genes and their Open Targets disease-association scores.

**Official gene symbol**	**log_2FC**	**Adjusted *P*-value**	**Direction of regulation**	**Disease-association score**
SH2D2A	1.797	1.79×10^{-27}	Upregulated	0.501
C1QTNF6	1.610	2.86×10^{-14}	Upregulated	0.541
NKX2-5	−1.107	1.64×10^{-7}	Downregulated	0.714
CD226	−1.768	1.86×10^{-5}	Downregulated	0.541
GLIS3	1.246	1.12×10^{-4}	Upregulated	0.642
DUOX2	−1.154	1.16×10^{-3}	Downregulated	0.808
PAX8	1.233	2.94×10^{-3}	Upregulated	0.785
STAT4	−1.014	2.82×10^{-2}	Downregulated	0.494
DUOXA2	−1.175	3.16×10^{-2}	Downregulated	0.719
PTPN22	1.593	3.73×10^{-2}	Upregulated	0.547

Note: Differential expression values were obtained from GSE158085, and disease-association scores were obtained from the Open Targets Platform for hypothyroidism (MONDO_0005420; version 26.06).

Table S2. Degree centrality and Maximal Clique Centrality (MCC) values of connected genes in the STRING protein–protein interaction network at a confidence threshold of 0.4.

**Gene**	**Degree**	**MCC**
**STAT4**	2	2
**PTPN22**	3	3
**PAX8**	4	4
**NKX2-5**	1	1
**GLIS3**	1	1
**DUOXA2**	2	2
**DUOX2**	2	2
**CD226**	2	2
**C1QTNF6**	1	1

Table S3. Complete transcription factor enrichment results obtained using X2K. Raw hypergeometric *p*-values were obtained from X2K, and adjusted *p*-values were calculated using the Benjamini–Hochberg false discovery rate (FDR) correction for multiple testing.

**Rank**	**Transcription factor**	**Raw hypergeometric *p*-value**	**BH-adjusted *p*-value**	**Enriched targets**
**1**	ERG	0.03675	**0.18186**	1
**2**	SUZ12	0.06062	**0.18186**	2
**3**	NANOG	0.14460	**0.28920**	1
**4**	TCF3	0.23470	**0.31410**	1
**5**	RUNX1	0.29350	**0.31410**	1
**6**	TP63	0.31410	**0.31410**	1

Table S4. Kinase enrichment results for the top 20 kinases. Raw hypergeometric *p*-values were obtained from X2K, and adjusted *p*-values were calculated using the Benjamini–Hochberg false discovery rate (FDR) correction across the complete kinase enrichment results (n = 135).

Rank	Protein kinase	Raw hypergeometric *p*-value	BH-adjusted *p*-value
**1**	HIPK2	3.871×10^{-9}	5.226×10^{-7}
**2**	CSNK2A1	8.228×10^{-7}	5.554×10^{-5}
**3**	CK2	1.503×10^{-6}	6.284×10^{-5}
**4**	MAPK1	1.862×10^{-6}	6.284×10^{-5}
**5**	GSK3B	5.552×10^{-6}	1.325×10^{-4}
**6**	ERK1	6.002×10^{-6}	1.325×10^{-4}
**7**	ABL1	6.871×10^{-6}	1.325×10^{-4}
**8**	CDK1	1.909×10^{-5}	3.221×10^{-4}
**9**	AKT1	2.398×10^{-5}	3.357×10^{-4}
**10**	MAPK14	2.487×10^{-5}	3.357×10^{-4}
**11**	CDK4	4.218×10^{-5}	5.177×10^{-4}
**12**	CK2ALPHA	4.981×10^{-5}	5.604×10^{-4}
**13**	ERK2	7.464×10^{-5}	7.751×10^{-4}
**14**	JNK1	1.164×10^{-4}	1.122×10^{-3}
**15**	MAPK3	2.003×10^{-4}	1.803×10^{-3}
**16**	CDK2	2.345×10^{-4}	1.979×10^{-3}
**17**	PKBALPHA	3.332×10^{-4}	2.646×10^{-3}
**18**	GSK3BETA	7.887×10^{-4}	5.693×10^{-3}
**19**	CK2A2	8.012×10^{-4}	5.693×10^{-3}
**20**	TGFBR2	1.216×10^{-3}	8.208×10^{-3}
